# Improving medication adherence to endocrine therapy in breast cancer patients: a mixed-methods systematic review of effective communication strategies for healthcare providers

**DOI:** 10.1016/j.breast.2025.104510

**Published:** 2025-05-28

**Authors:** M.A.A. Smits, L.H. Mammatas, L. Schoonhoven, S.C.J.M. Vervoort

**Affiliations:** a Department of Oncology and Breast Cancer Centre, Reinier de Graaf Hospital, Reinier de Graafweg 5, 2625 AD, Delft, the Netherlands; b Department of General Practice & Nursing Science, Julius Center for Health Sciences and Primary Care, University Medical Center Utrecht, Utrecht University, Universiteitsweg 100, 3584 CG, Utrecht, the Netherlands; c School of Health Sciences, Faculty of Environmental and Life Sciences, University of Southampton, University Road, Southampton, SO17 1BJ, United Kingdom

## Abstract

**Background:**

Breast cancer is the most common cancer in women worldwide. Adjuvant endocrine therapy (AET) improves survival, but adherence often declines, increasing mortality risk. While previous research has focused on technological and educational approaches to reduce this decline, this review examines the role of effective communication in improving medication adherence in breast cancer patients

**Aim:**

This review explores key aspects of effective communication to enhance AET adherence in breast cancer patients, offering strategies for healthcare providers (HCPs) to better support patients in managing their treatment.

**Design:**

A mixed-methods systematic review was conducted following the Joanna Briggs Institute (JBI) approach to qualitative synthesis, reported according to the PRISMA statement. Registered in PROSPERO (#CRD42024480202).

**Data sources:**

A search in PubMed, EMBASE, CINAHL, Psycinfo, Cochrane and Web of Science identified studies on HCP–patient communication and medication adherence in breast cancer patients using AET. The quality was assessed using JBI tools.

**Results:**

Fifteen studies (one mixed-methods, five qualitative, six quantitative and three (systematic) reviews/meta-analysis) were included. Three synthesized findings were identified: 1) Content of communication (discussions on the importance and necessity of AET and adherence, AET-related side effects, and strategies to adhere to AET); 2) Process of communication (building a trusting relationship, fostering open and mutual communication, engaging patients, and aligning with patients); and 3) Contextual preconditions (time constraints and ongoing support).

**Conclusion:**

Effective communication between HCPs and patients is vital for improving medication adherence. Training HCPs and providing consistent support, especially from nurses, enhances patient care and quality of life.

## Highlights

• Women feel the need to initiate conversations about adjuvant endocrine therapy.
• Building a trusting relationship is vital for open and mutual communication.
• Contextual preconditions for communication are sufficient time and ongoing support.
• Adherence communication fits in nursing care due to nurses' ongoing patient support.

## 1 Introduction

Breast cancer is the most frequent cancer among women worldwide, with a lifetime risk of 1 in 11 in Europe [[1], [2], [3]]. Adjuvant endocrine therapy (AET) is widely used, as 70 % of breast cancers are hormone receptor-positive resulting in oestrogen driven cell division and spreading [4,5]. AET inhibits binding of oestrogen to hormone receptor-positive breast cancer cells and can significantly improve survival [6]. AET requires daily use for five up to ten years, but adherence is often suboptimal. Various studies report that non-adherence rates to AET may reach approximately 50 %. Adherence declines from 90 % in the first to 77 % in the third year and drops to 51 % by the fifth year [[7], [8], [9], [10]]. Optimal AET use is essential, as non-adherence increases disease-related mortality [7].

Understanding the factors contributing to this decline in adherence is crucial. Medication adherence is defined as "the degree to which the person's behaviour corresponds with the agreed recommendations from a healthcare provider" [11]. It encompasses timely and consistent medication intake and the patient's engagement with the prescribed treatment plan. Barriers related to adherence to AET are patient-related (e.g., beliefs, fear of adverse effects), therapy-related (e.g., side effects, lifestyle disruption), healthcare system-related (e.g., patient/provider relationships), socioeconomic (e.g., medication costs and racial disparities in adherence), and disease-related factors (e.g., comorbidities, cancer stage) [[12], [13], [14]]. Recognizing and addressing the multifaceted barriers to adherence can lead to improved patient outcomes and reduced mortality rates [15,16].

Previous research on improving AET adherence in breast cancer patients focused on technological and educational interventions, such as symptom management, patient education, cost-lowering policy changes, survivorship programs, websites, information folders and apps, and text- and verbal-based interventions [[17], [18], [19], [20], [21], [22], [23], [24], [25], [26], [27]]. While these approaches have been valuable, recent findings highlight that difficulties in interacting and communicating with the medical team are significantly associated with non-persistence [28], underscoring the crucial role of healthcare provider (HCP)-patient interactions in adherence [29]. In addition, adherence to AET is often suboptimal among breast cancer patients, as communication strategies frequently lack consistency and a structured approach. For instance, regular communication about the importance of medication adherence was reported by only 44.2 % of patients, while 33 % reported that the topic was mentioned only a single time [30]. Patients also report receiving insufficient information and support from HCPs [31,32]. Moreover, a good HCP–patient relationship was associated with better adherence, although these results remain tentative [33]. HCP–patient communication influences patients' beliefs about their treatment, with greater concerns leading to poorer communication and stronger beliefs in the treatment's necessity, resulting in more positive communication [34].

Effective communication between HCPs and patients is a fundamental element of care, essential for achieving successful healthcare outcomes [35]. Currently, there is limited detailed insight into what effective communication entails to improve medication adherence in breast cancer patients. This highlights the need to better understand effective communication between women using AET and HCPs and how it might affect medication adherence, alongside the importance of technological and educational efforts. Therefore, this review aims to explore key aspects of effective communication to enhance medication adherence to AET in breast cancer patients, offering strategies for HCPs.

## 2 Methods

### 2.1 Design

We followed the Joanna Briggs Institute (JBI) methodological guidance for mixed-methods systematic reviews [36]. An integrative mixed-methods design was used to synthesize any possible qualitative and quantitative findings [36]. Furthermore, other documents or reports could also be part of the review when appropriate.

This review was reported following the Preferred Reporting Items for Systematic Reviews and Meta-analysis (PRISMA) framework to enhance transparent reporting [37]. The study protocol was registered in Prospero (registration number # CRD42024480202). No ethical approval was required.

### 2.2 Databases and searches

Electronic searches were conducted in MEDLINE via PubMed, PsycINFO, Cochrane Library, CINAHL, Embase, and Web of Science from February 2023 to June 2023 and updated in December 2024.

Continuous assessment and refinement were essential in developing the search strategy, as this process is iterative [38]. Each information database was searched using MeSH-terms, CINAHL headings, the PsycINFO thesaurus and keywords. The main keywords used were medication adherence, communication and endocrine therapy. The search terms were combined with the Boolean operators ‘AND’ and ‘OR’. No filters or time restrictions were applied.

Table 1 shows the search strategy for PubMed, which was similarly applied to other databases with necessary modifications. The reference lists of the included articles were screened for additional relevant studies. In December 2024, the search was repeated in all relevant databases, but no new articles met the inclusion criteria.

**Table 1:**
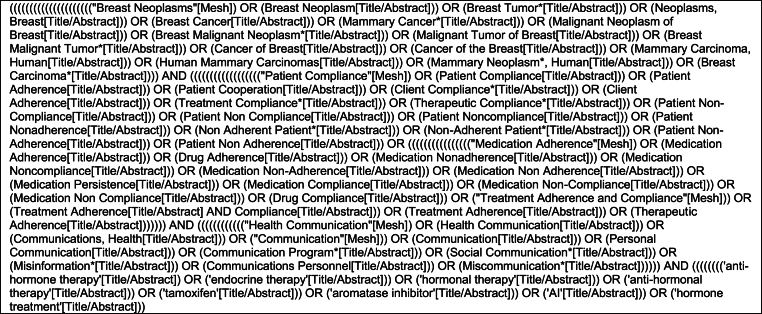
Search strategy PubMed (conducted on 12-6-2023, and 13-12-2024).

### 2.3 Eligibility criteria and study selection

A study was eligible if it met the following criteria.

1. The outcome was the role of HCP-patient communication in AET adherence;
2. The study population included breast cancer patients using AET;
3. The studies were written in English or Dutch.

Three researchers (MS, SV, and LM) independently screened titles and abstracts using Rayyan [39] to identify relevant studies. They then independently performed full-text screening based on the eligibility criteria. The researchers resolved disagreements, and when they did not reach a consensus, they discussed this with the research team.

### 2.4 Data extraction

The data, which were extracted using the JBI mixed-methods extraction tool, encompasses a comprehensive range of elements, including reviewer details, publication information, study characteristics, and key findings [40]. Three reviewers (MS, SV, and LM) extracted the data, and the research team reviewed the tabled extractions for completeness and accuracy.

### 2.5 Quality appraisal

Three reviewers (MS, SV and LM) assessed the quality of the included studies using the JBI Critical Appraisal Checklist [41].

The researchers (MS and SV, and MS and LM) independently appraised the individual components of the checklists by indicating whether a study did (+1) or did not (+0) adhere to the quality requirements, or whether this was unclear (+0.5). A score of 0 was also assigned to an item when information was not provided. Finally, a summary score was calculated for each study. The maximum final score for qualitative studies was 10; for quantitative studies 8; and for systematic reviews (SR) and meta-analysis 11. The quality appraisal did not impact study inclusion, as lower-scoring studies could still provide valuable insights.

### 2.6 Data transformation and synthesis

This review followed a convergent integrated approach using the JBI methodology for mixed-methods systematic reviews [41]. Therefore, three steps were performed to analyze the data. First, all qualitative and quantitative findings relevant to the aim were extracted. The quantitative findings were ‘qualitized’ into a textual description, providing a narrative interpretation of each quantitative finding. In addition, we provide insight into the different study designs, including the number of articles per design and, where relevant, illustrative quantitative quotes. Second, all findings were reread to gain a thorough understanding of the data, then the data was categorized into categories with similar meanings. Third, these categories were thematically aggregated into synthesized findings based on similarity in meaning. NVivo14 was used to assist the analysis [42]. Two reviewers (MS and SV) performed the data transformation and synthesis. The categories and synthesized findings were discussed with the research team.

## 3 Results

### 3.1 Search and selection strategy

**Fig. 1:**
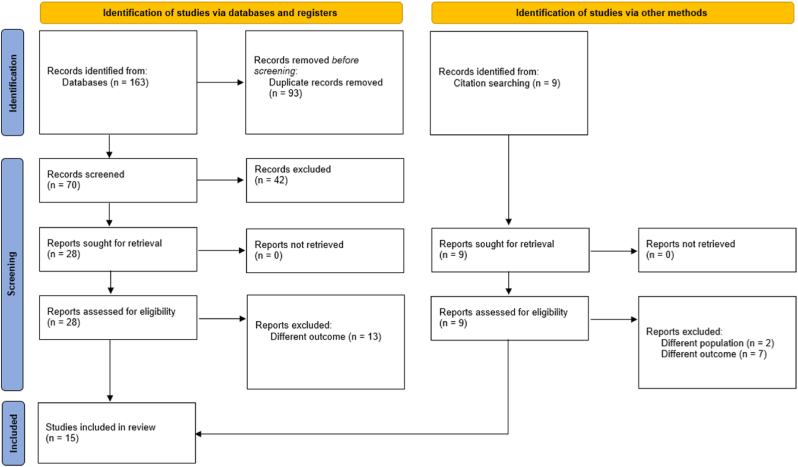
PRISMA flow-chart [1].

The search identified 163 studies, of which 93 were duplicates. After screening based on title and abstract, 42 studies were excluded. 28 studies were assessed for eligibility, and 15 were included in this review. See Fig. 1 for the PRISMA flowchart.

### 3.2 Study characteristics

Five qualitative studies, six cross-sectional studies, one mixed-methods study and three (systematic) reviews and meta-analyses were included. The study characteristics are per design presented in Table 2, Table 3, Table 4, Table 5. The qualitative studies involved one focus group, one linguistic analysis, and three semi-structured individual interviews. All quantitative studies (n = 6) were cross-sectional and used descriptive statistics and/or regression analyses. The mixed-methods study used a questionnaire and semi-structured interviews. Of the three reviews, one was a systematic review, one was a narrative review, and one was a meta-analysis. Most studies were conducted in the USA (n = 9), Canada (n = 2) and Germany (n = 2). In total, 6823 patients (4698 patients from one meta-analysis article) and 69 HCPs participated.

**Table 2:** Study characteristics of the qualitative studies.

Author, year, country	Data collection and data analysis	Sample size and type of participants	Outcome	Study quality^a^
Davidson, 2007, USA	Record patient-oncologist interactions and conduct separate post-visit interviews Linguistic analysis	N = 14 for HCP N = 28 for patients	Oncologist-patient discussions about adjuvant hormone therapy were generally good but did not address potential difficulties of remaining adherent with long-term therapy.	5
Farias, 2015, USA	Individual semi-structured interviews Qualitative principles of inductive reasoning	N = 22 patients	[1] information exchange, [2] decision-making to take and continue adjuvant endocrine therapy ((A)ET), [3] enabling patient self-management monitoring potential side effects, and [4] emotional support.	7
Lambert, 2018, Canada	Individual interviews Inductive thematic and constant comparative analysis	N = 22 patients	The decision-making process around AET persistence became a balancing act between quality of life and quantity of life.	9.5
Roche, 2023, France	Focus group interviews Thematic analyses using a general inductive approach	N = 17 for HCP N = 28 for patients	‘Representations on adjuvant ET’ and ‘relationship between patients and healthcare providers’	8
Toivonen, 2021, Canada	Semi-structured individual interviews Thematic analysis using an inductive, descriptive approach	N = 9 for HCP N = 38 for patients, n = 23 for persisted and n = 15 for non-persisted	The considerable overlap in themes among the three groups suggests broad recognition of salient factors relevant to AET use and that associated strategies to improve use may be acceptable to patients and providers alike.	9

a JBI Critical Appraisal Checklist for Qualitative Research.

**Table 3:** Study characteristics of the cross-sectional studies.

Author, year, country	Data collection and data analysis	Sample size and type of participants	Outcome	Study quality^a^
Arriola, 2014, USA	Survey Regression and mediation analyses	N = 200 patients	Necessity beliefs mediated the relationship between frequency of physician communication and medication adherence (necessity beliefs b = 0.18, p < 0.05; physician communication b = 0.13, p > 0.05). There was no evidence of medication concerns mediating the relationship between frequency of physician communication and medication adherence.	7
Kimmick, 2015, USA	Survey Regression analyses	N = 112 patients	The presence of symptoms (p = 0.03) and lower self-efficacy for physician communication (p = 0.009) were associated with higher levels of intentional non-adherent behaviors.	8
Lin, 2013, USA	Survey Multivariate logistic regression analyses	N = 100 patients	Despite very high communication scores, one-third of participants reported they had not discussed side effects with providers. Multivariate analysis showed that after controlling for age, education, race and medication beliefs, women who had difficulty asking providers for more information were more likely to report side effects (odds ratio 8.27, 95 % confidence interval 1.01–69.88).	8
Liu, 2013, USA	Survey Bivariate and multivariate logistic regression analyses	N = 921 patients	After controlling for potential confounders, greater patient-centred communication from oncologists at 18 months post-diagnosis positively predicted ongoing use of hormone therapy at 36 months after BC diagnosis (AOR = 1.22, P = 0.006). *(…)* Patients with greater self-efficacy in patient-physician interactions were also more likely to adhere to hormone treatment (AOR = 1.04, P = 0.04).	7
Rosso, 2023, Italy	Survey Descriptive statistics	N = 373 patients	Eighty-eight per cent of patients who experienced AET side effects reported talking about it with the gynecologist during follow up visits. Overall, 77 % of patients reported that the gynecologist asked them first about side effects and therapy compliance. *(…)* Only 9 % of patients who experienced menopausal symptoms made regular visits to the dedicated menopause service of the Breast Unit. Fifteen per cent of patients received psychological support from the dedicated psychology service of the Breast Unit, and ninety-four per cent of them reported that it was beneficial and that they would have recommended it to other women diagnosed with breast cancer. Overall, 94 % of women felt well supported during follow-up visits and reported being correctly informed by gynecologists about adverse side effects of AET. On the other hand, 12 % of patients who discontinued treatment reported that they would have continued it if they were better informed about side effects and possible therapies to control them.	5
Wuensch, 2015, Germany	Survey Descriptive analyses	N = 281 patients	The decision for or against anti-hormonal therapy was made alone by 8.9 % of participants. In 45.6 % of cases, physician and patient decided together, and in 37.4 %, the physician alone made the decision. *(…)* Information regarding effects has no impact, whereas patients who got information on side effects have significantly better adherence. *(…)* Patients who got information on the possibilities of supportive therapy in the case of side effects tend to have a better adherence than those who did not. While the comprehensiveness of information generally has no influence, getting detailed answers to questions leads to significantly better adherence (p = 0.014).	6.5

a JBI Critical Appraisal Checklist for Analytical Cross-Sectional Studies.

**Table 4:** Study characteristics of the mixed-methods study.

Author, year, country	Data collection and data analysis	Sample size and type of participants	Outcome	Study quality^a^,^b^
De Mendoza, 2019, USA	Semi structured interviews and survey Consensual Qualitative Research framework and descriptive analyses	N = 10 for interviews (HCP) N = 19 for surveys (HCP)	Several providers reported that their patients concealed important adherence information (e.g., skipped doses), so as not to disappoint them. When patients disclose medication interruption, they usually wait until the in-person visit, creating time lags in communication. Participants' suggestions to reduce the delay between discontinuation and disclosure emphasized more frequent contact between visits but added that they lacked the resources to do so. Another challenge in communication was asking the right questions. Strategies to obtain more accurate information included asking more precise questions (e.g., have you been taking the medication – every day/as prescribed) and building trust with patients. Participants also described the role of patient navigators, nurses, and pharmacists as crucial because some patients are more likely to open up and share their concerns with them rather than the doctors. Most providers assessed adherence during their patients' regular in-person check-ups (84.2 %) and via patient self-report (78.9 %). Assessing discontinuation was perceived to be moderately challenging (M = 3.74, SD = 2.16-range 1–7). The most salient challenges providers reported into assessing adherence were patients' delay in informing about discontinuation (78.9 %) and over-reporting adherence (57.9 %).	8.5^a^, 4^b^

a JBI Critical Appraisal Checklist for Qualitative Research.

b JBI Checklist for Analytical Cross-Sectional Studies.

**Table 5:** Study characteristics of the systematic reviews and meta-analyses studies.

Author, year, country	Aim	Sample size of articles	Outcome	Study quality^a^
Finitsis, 2019, USA	To [1] quantitatively review the aggregate effect of interventions designed to optimize AET adherence within the current literature and [2] meta-analyze these effects across studies' by intervention design	N = 7 studies (total of 4698 patients included)	An overall null effect across all interventions (k = 8; d [95 % CI] = 0.28 [−0.05, 0.61]); however, sensitivity analyses showed that interventions that used bi‐directional communication showed statistically significant effects relative to control groups within each study (k = 4; d [95 % CI] = 0.59 [0.23, 0.95]) while those relying only on providing information to the patient (one‐way communication) did not (k = 4; d [95 % CI] = −0.03 [−0.27, 0.20]).	10
Hadji, 2010, Germany	To evaluate the data regarding compliance and persistence to adjuvant endocrine therapy and presents strategies for their improvement.	Unknown	Promising strategies for enhancing adherence to treatment in clinical practice include improving access to healthcare, increasing patient satisfaction, managing side effects, patient education, and better communication between the patient and healthcare provider. Positive relationships between patients and their healthcare providers and frequent monitoring and feedback may be most effective.	5.5
Miaskowski, 2008, USA	To summarize the issues surrounding adherence, paying specific attention to adjuvant endocrine therapy for breast cancer, and outlines strategies to reduce non-adherence that nurses can incorporate into clinical practice.	Unknown	Nurses can positively effect patient outcomes when they recognize and manage non adherence issues. Continuity of healthcare and consistency of dialogue between patients and caregivers are essential for improving adherence; the key is finding a way to reach all patients, especially in underserved populations, for regular communication and follow-up.	5

a JBI Critical Appraisal Checklist for systematic reviews and meta-analysis studies.

#### 3.2.1 Quality appraisal

Table 6, Table 7, Table 8, Table 9 show the quality appraisal of the included studies. The mean quality score for the quantitative studies was 6.9/8 (range: 5/8 and 8/8), and for the qualitative studies, it was 7.7/10 (range: 5/10 and 9.5/10). The mean score for the mixed-methods study was 12.5/18. Lastly, the mean quality score for SR and meta-analysis studies was 6.8/11 (range: 5/11 and 10/11).

**Table 6:**
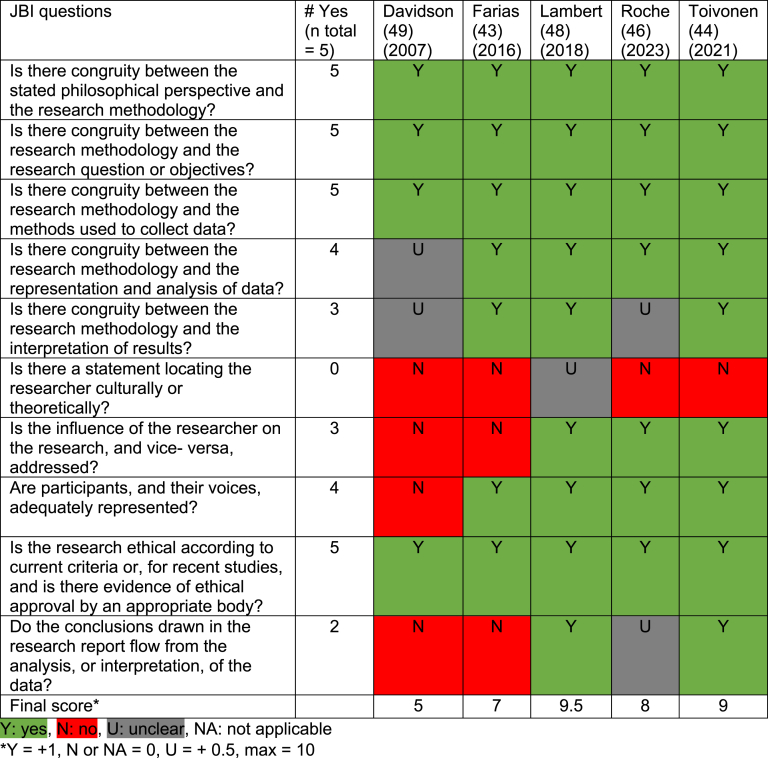
Quality appraisal of qualitative studies.

**Table 7:**
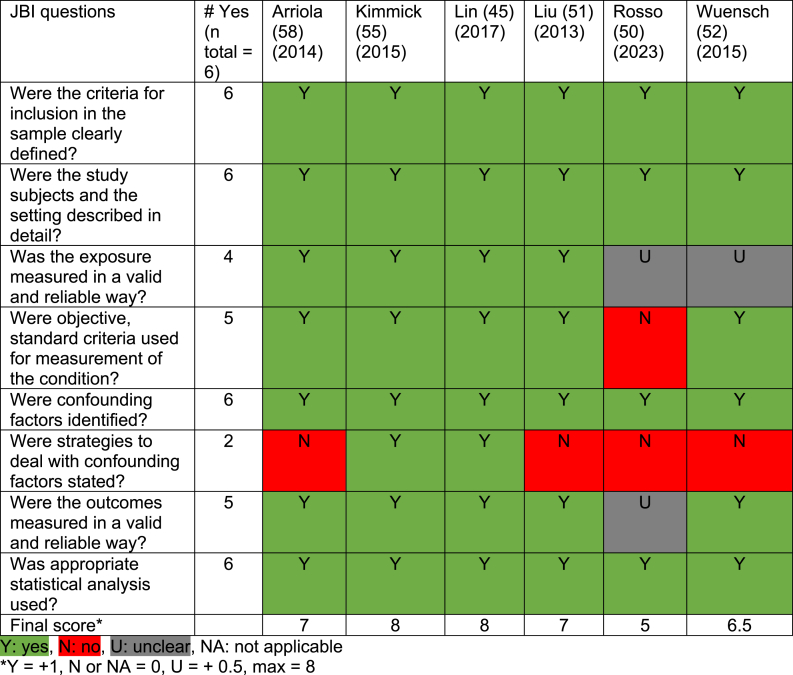
Quality appraisal of cross-sectional studies.

**Table 8:**
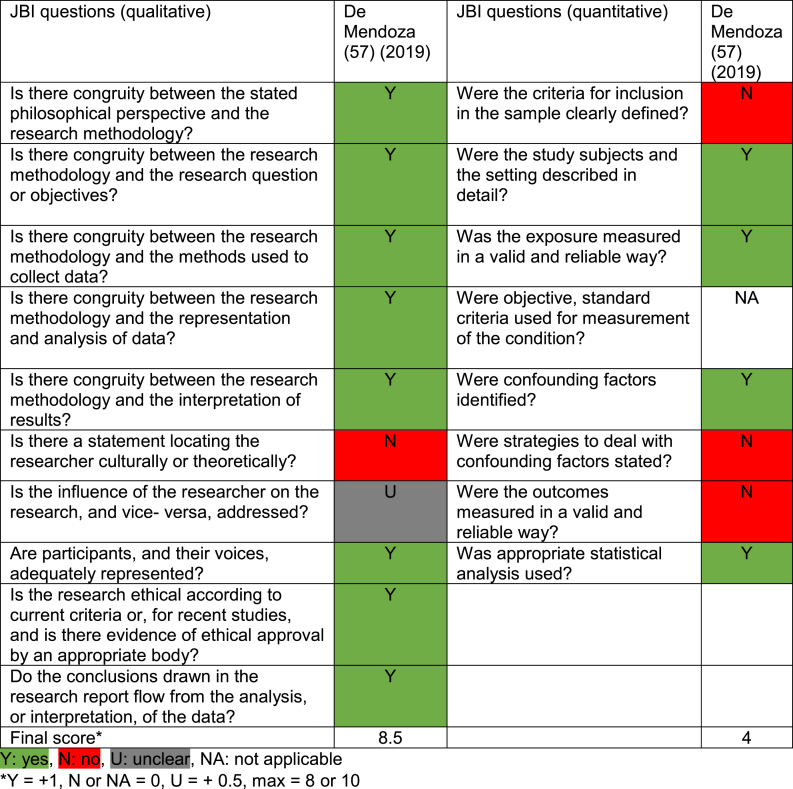
Quality appraisal of mixed-method study.

**Table 9:**
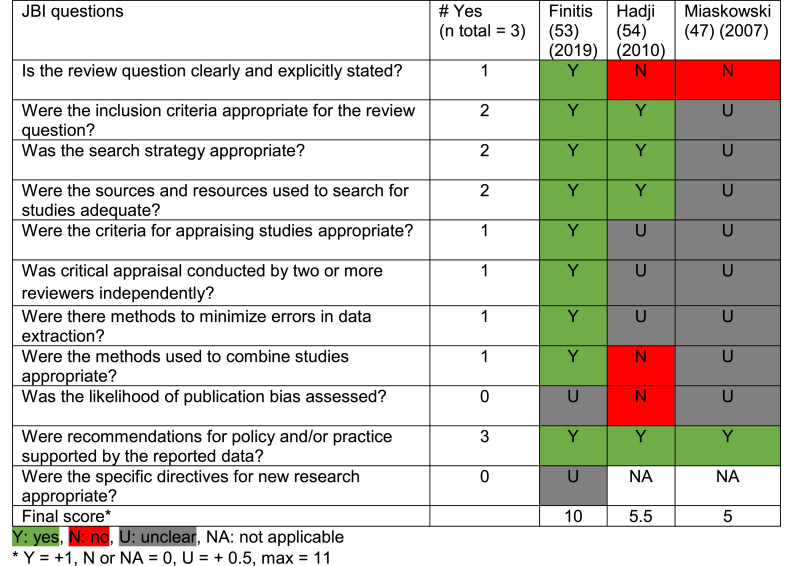
Quality appraisal of systematic reviews and meta-analysis studies.

In most qualitative studies, congruity between research question, methods and analysis was sound. A statement locating the researcher culturally or theoretically was lacking. In cross-sectional studies, the strategies to address confounding factors were not stated or were lacking in five of the seven studies. However, in all studies, confounding factors were identified. The mixed-methods study did not show aggregated results, but these were presented separately, and the quantitative components mainly resulted in unclear JBI answers. Lastly, the SR and meta-analysis studies were not well-executed SR's, resulting in multiple unclear answers to the JBI questions.

### 3.3 Data synthesis

**Fig. 2:**
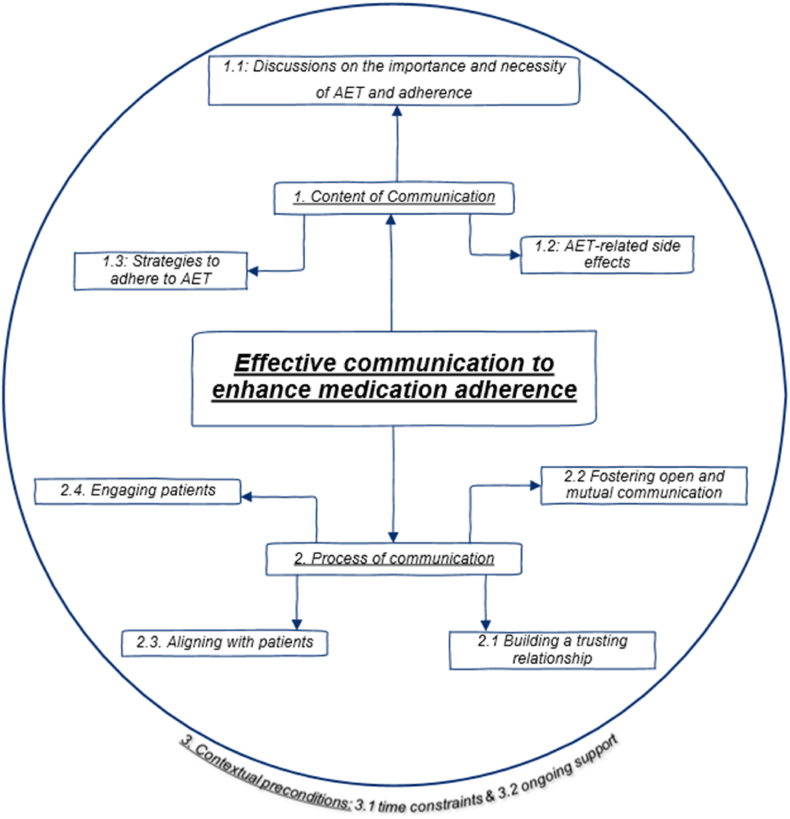
Overview of the findings. The figure illustrates the interrelationships among the findings, highlighting their distinct yet interconnected roles in the overall process. Specifically, Finding 3 represents the contextual preconditions essential for enabling and supporting the successful implementation of Findings 1 and 2. As such, Finding 3 is depicted outside the central circle in the figure, symbolizing its overarching influence on the entire framework and highlighting its key role in creating the necessary conditions for the successful implementation of the other findings.

Our systematic review revealed three synthesized findings: 1) Content of communication, with three subcategories: discussions on the importance and necessity of AET and adherence, AET-related side effects, and strategies to adhere to AET; 2) Process of communication, with four subcategories: building a trusting relationship, fostering open and mutual communication, engaging patients, and aligning with patients; and 3) Contextual preconditions for communication, with two subcategories: time constraints and ongoing support. These findings emerged from 27 categories based on 78 unique findings and 213 quotes: 28 quantitative and 185 qualitative quotes. Each category could contain multiple unique findings from one study. An overview of the findings is visually summarized in Fig. 2. In addition, the synthesized findings are supported by key quotes. Table 10 provides an overview of these quotes, indicating which articles support what finding, including the study design of those articles and the first author. Where relevant, the findings are further supported by corresponding quantitative data.

**Table 10:** Overview table of the synthesized findings, number of articles and design including first author and key-quotes and quantitative data and their statistical significance (when relevant).

Synthesized Finding	# of articles and design	1st author	Key-quotes	Quantitative data and their statistical significance (when relevant)
**Synthesized finding 1: Content of communication in relation to adherence**				
*Synthesized finding 1.1: Discussions on the importance and necessity of AET and adherence*	Total: 7 # cross-sectional: 1 # qualitative: 5 # (systematic) review: 1	Farias [43], Toivonen [44], Lin [45], Roche [46], Miaskowski [47], Lambert [48], Davidson [49]	*“Beliefs about the necessity of AET were largely influenced by how it was positioned in discussions with HCPs as an essential and expected step in the treatment trajectory* [48]*.”* *“No discussion took place explicitly linking adherence to “best outcomes” of therapy, and no oncologist discussed what to do if a dose was missed or asked patients if they foresaw any problems with adhering to therapy* [49]*.”*	Of those who did discuss side effects with a medical provider, 87 % reported that they initiated the conversation, (…) [45].
*Synthesized finding 1.2: AET-related side effects*	Total: 6 # cross-sectional: 3 # qualitative: 2 # (systematic) review: 1	Roche [46], Miaskowski [47], Lambert [48], Rosso [50], Liu [51], Wuensch [52]	*“A breakdown in the patient-HCP relationship, however, damaged women's trust in their physician, resulting in a perceived lack of support, (…). Some women influenced their decision not persist with AET. (…) Access to other HCPs, such as nurses (…), provided a trusted, and often more accessible, resource for women* [48]*.”* *“Eighty-eight* per *cent of patients who experienced AET side effects reported talking about it with the gynecologist during follow up visits* [50]*.”*	In follow-up visits, 88 % of patients who experienced side effects from AET reported discussing these issues with their HCP [50]. While general information about treatment effects may not significantly impact adherence, patients who receive detailed information about side effects and HCPs providing specific answers to patient questions demonstrate significantly higher adherence rates (p = 0.014) [51,52].
*Synthesized finding 1.3: Strategies to adhere to AET*	Total: 6 # cross-sectional: 1 # qualitative: 3 # (systematic) review: 2	Farias [43], Toivonen [44], Roche [46], Miaskowski [47], Rosso [50], Finitsis [53]	*“These typically included information about breast cancer, the benefits of AET, possible side effects of AET, what to do when these occur, and content addressing lifestyle enhancements (diet, exercise)* [53]*.”* *“Physicians also shared how they would choose to present information related to adjuvant ET, in a way that it is easily understood by their patients* [46]*.”*	This is important as 12 % of patients who discontinued treatment would have continued if they had been better informed about side effects and management options [50].
**Synthesized finding 2: Process of communication in relation to adherence**				
*Synthesized finding 2.1: Building a trusting relationship*	Total: 5 # cross-sectional: 2 # qualitative: 2 # (systematic) review: 1	Toivonen [44], Lambert [48], Rosso [50], Liu [51], Hadji [54]	*“In cancer care in particular, empathy and understanding lead to greater satisfaction and adherence to treatment, of which the tone of the physician-patient relationship is particularly important* [54]*.”* *“Women who perceived their physicians as empathic, responsive, accessible, and knowledgeable about AET and had a positive relationship with their physicians and had a high level of trust and confidence in their recommendations were more inclined to discuss AET concerns in consults, seek help in managing side effects, and persist with AET* [48]*.”* *“It's not a one-size-fits-all. It's a communication and consultation process with each individual patient, depending on their disease, characteristics, goals, beliefs, comorbidities and age (44.).”*	
*Synthesized finding 2.2: Fostering open and mutual communication*	Total: 4 # cross-sectional: 1 # qualitative: 3	Toivonen [44], Roche [46], Davidson [49], Kimmick [55]	*“Participants noted that their interactions with HCPs, particularly oncologists, mattered and were important enough to influence their decision to persist with AET* [44]*.”*	Additionally, lower self-efficacy in physician communication was significantly associated with higher levels of intentional non-adherence (p = 0.009) [55].
*Synthesized finding 2.3: Engaging patients*	Total: 4 # qualitative: 2 # (systematic) review: 2	Toivonen [44], Roche [46], Finitsis [53], Hadji [54]	*“Communication needs to be reciprocal to be effective* [44]*.”* *“Health care providers should encourage patients to express their concerns and ask questions, and they should respond empathically to patients' emotions* [54]*.”* *“HCPs emphasized providing a balanced perspective about the risks and benefits of AET use and encouraged patients' autonomy in decision-making based on all available information* [44]*.”*	
*Synthesized finding 2.4: Aligning with patients*	Total: 7 # cross-sectional: 1 # qualitative: 3 # (systematic) review: 2 # mixed-method: 1	Farias [43], Toivonen [44], Lin [45], Roche [46], Miaskowski [47], Hadji [54], de Mendoza [57]	*“Moreover, most HCPs admitted that they don't necessarily have the training to adequately assist patients facing psychological difficulties over adjuvant ET* [46]*.”* *“Better patient education and the earlier management of side effects may increase tolerability and avert the discontinuation of therapy for breast cancer* [54]*.”* *“Improved patient understanding of the actual recurrence risk also is needed, so that women do not over- or underestimate this risk and can make more informed decisions about the risk/benefit profile of their treatment* [54]*.”*	
**Synthesized finding 3: Contextual preconditions in relation to adherence**				
*Synthesized finding 3.1: Time constraints*	Total: 2 # qualitative: 2	Toivonen [44], Roche [46]	*“One participant reported that her frustration with feeling ignored and very brief appointment times caused her to stop consulting with her oncologist about AET* [44]*”.*	
*Synthesized finding 3.2: Ongoing support*	Total: 6 # cross-sectional: 1 # qualitative: 2 # (systematic) review: 2 # mixed-method: 1	Roche [46], Miaskowski [47], Lambert [48], Hadji [54], de Mendoza [57], Arriola [58]	*“Some women experienced a lack of continuity of care when transitioning from oncology to primary care. (…) Women who continued to see an oncologist reported greater satisfaction with the provision of follow-up care as well as a sense of safety and confidence* [48]*.”* *“A lack of access to timely follow-up care meant some women felt abandoned during the survivorship period and (…) influenced their decisions to stop AET early* [48]*.”* *“All participants agreed on the importance of consistency with an interlocutor who would be the same throughout their care path* [46]*.”* *“The role of the oncology nurse is paramount in augmenting patient-provider interaction* [47]*.”* *“Frequently monitoring the patient and providing feedback may be the most effective way to improve adherence* [54]*.”*	Frequent and well-structured physician communication significantly improves medication adherence (p < 0.05.) [58].

#### 3.3.1 Synthesized finding 1: content of communication in relation to adherence

In our review, the content of communication concerning adherence includes discussions on the importance and necessity of AET, its purpose, (management of) side effects, and strategies to improve adherence to AET.

#### 3.3.2 Synthesized finding 1.1: discussions on the importance and necessity of AET and adherence

Women reported that their HCPs discussed AET medication's purpose, benefits, and duration [43]. Additionally, there were discussions about the benefits and potential side effects [43,44]. However, among patients who discussed side effects with an HCP, a significant majority (87 %) initiated the conversation themselves [45]. Hadji recommends that oncologists should address the issues of adverse events and adherence to therapy at every patient visit, specifically highlighting the risk of breast cancer recurrence and the benefits of AET. When patients do not fully grasp the necessity of AET, they may be less inclined to adhere to the prescribed regimen [46]. Misunderstandings about why and how to take medications can undermine a patient's confidence in their treatment plan [47]. Moreover, patients' beliefs about the necessity of AET are strongly influenced by how HCPs present it [48]. Furthermore, critical aspects of adherence were often overlooked, such as guidance on what to do if a dose was missed or discussions regarding potential challenges with adhering to AET [49]. Physicians also rarely asked whether patients foresaw any problems with long-term therapy adherence, leaving patients feeling unsupported regarding these practical and emotional challenges.

#### 3.3.3 Synthesized finding 1.2: AET-related side effects

In follow-up visits, 88 % of patients who experienced side effects from AET reported discussing these issues with their HCP [50]. HCPs often provide a broad explanation of what AET is and why it is recommended but may underestimate the burden of AET-related side effects, particularly when those side effects are not life-threatening but significantly impact the quality of life. When physicians ignore or downplay these side effects and withhold certain treatment details, it will discourage patients from reporting their struggles with treatment tolerance, increasing the risk of non-adherence [46,47].

While general information about treatment effects may not significantly impact adherence, patients who receive detailed information about side effects and HCPs providing specific answers to patient questions demonstrate significantly higher adherence rates (p = 0.014) [51,52]. In addition, women were hesitant to ask their physicians about AET side effects because they feared their concerns would be met with resistance or apathy or dismissed as being insignificant. Feelings of discomfort, especially related to discussing intimate and sexual life, and lack of support from doctors were noted as barriers to effective communication [46,48]. In contrast, nurses and pharmacists can serve as trusted, often more accessible sources of support for women [48].

#### 3.3.4 Synthesized finding 1.3: strategies to adhere to AET

Finitsis et al. addressed the benefits and side effects of AET and provided advice on managing them and improving lifestyle enhancements [53]. This is important as 12 % of patients who discontinued treatment would have continued if they had been better informed about side effects and management options [50].

HCPs identified education as a primary tool in supporting AET adherence, including proactive discussion of side effects [44]. Physicians emphasized presenting information about AET in a way that is easily understandable for patients [46], and all women in the study were able to explain how the drugs worked [43]. Nurses can be key in managing adverse events by talking with patients and informing other HCPs about potential issues [47].

#### 3.3.5 Synthesized finding 2: process of communication in relation to adherence

In our review, the communication process in the interaction between HCPs and patients with AET encompasses building a trusting relationship, fostering open and mutual communication, engaging patients and aligning with patients.

#### 3.3.6 Synthesized finding 2.1: building a trusting relationship

A trusting relationship between patients and HCPs is important for communication and medication adherence among patients with AET. This relationship includes maintaining a non-judgmental stance, supporting the patient, showing empathy, building rapport, and respecting patient perspectives. HCPs who promote this contribute to a positive relationship and facilitate open, patient-centred communication, leading to ongoing use of AET through a tailored approach [44,48,50,51,54]. Conversely, women expressed frustration when they felt that HCPs did not listen to them, were interpersonally cold, dismissed their concerns, or spent little time with them during appointments. This can lead to stopping consulting the oncologist about AET [44].

#### 3.3.7 Synthesized finding 2.2: fostering open and mutual communication

Open and mutual communication between HCPs and patients is essential for medication adherence in women using AET. Such communication involves a clear, honest, and transparent exchange of information where both parties feel comfortable sharing their thoughts, concerns, and questions. HCPs need to encourage open communication and ask open-ended questions in a non-judgmental manner [44]. This is important for the smooth running of treatment and could endanger it when both dynamics collide [46]. Open communication may result from the emotional intensity of the oncologist-patient relationship [49]. Additionally, lower self-efficacy in physician communication was significantly associated with higher levels of intentional non-adherence (p = 0.009) [55]. Synthesized finding 2.3: Engaging patients.

Engagement involves bidirectional information exchange, where HCPs and patients actively share and receive information. This approach empowers patients by involving them in their care and ensures that they feel heard and valued. Engaging patients in discussions, practising open communication, and being responsive to their concerns were highlighted as essential practices for fostering a supportive and collaborative environment [44,53,54]. Although managing side effects can be challenging, nearly half of the women who persisted with treatment did so because their HCPs or other survivors informed them. This expectation helped them mentally prepare and reduced the emotional impact of side effects [44].

Hadji emphasizes focusing on patients’ individual choices rather than just clinical study results and stresses how side effects of each therapy can be proactively identified, managed, and alleviated. Toivonen et al. agree that providing a balanced view of AET risks and benefits while encouraging patient autonomy in decision-making is essential. The importance of shared decision-making is underscored by the fact that some patients who discontinue AET do so without discussing their decision with their physician, possibly to avoid confrontation or discomfort [46].

#### 3.3.8 Synthesized finding 2.4: Aligning with patients

As an aspect of patient-centred care, aligning with patients involves tailoring communication, decisions, and treatment plans to reflect the patient's needs, values and goals [56]. Alignment helps create a clear, unified understanding and supports effective decision-making and communication. Aligning information with patient needs, using language similar to that used by their patient in simple, non-intimidating terms and employing effective questioning techniques are essential to enhancing communication competencies [54]. In addition, HCPs may upset patients who receive conflicting information about AET from different sources, highlighting the importance of coordinating care teams [57]. Training HCPs in communication skills and aligning information can improve patient interactions [46,54,57]. Effective training could also include assessing patients' preferred learning styles to ensure that information is conveyed in the most accessible and comprehensible manner [47]. By doing so, educating patients can significantly improve compliance with their medication [54].

On the other hand, women mentioned they want to be more informed about AET. They often made decisions about taking AET based on trade-offs with the risk of breast cancer recurrence and side effects from treatment. Therefore, they must be well-informed in making these decisions [43,44,54]. In addition, alignment on discussion topics appears to be important in treatment conversations, as HCPs tend to focus more on side effects, benefits, and treatment options, while patients prioritize understanding their prognosis, the risks and benefits of AET, and its long-term effects [54]. This underscores the importance of providing information and ensuring patients feel comfortable and empowered to ask for the necessary details [45].

#### 3.3.9 Synthesized finding 3: Contextual preconditions in relation to adherence

Our review identified contextual preconditions as essential factors for discussing AET and adherence. Two categories, *time constraints* and *ongoing* support, formed this finding.

#### 3.3.10 Synthesized finding 3.1: Time constraints

HCPs lack time during appointments, and it is often not feasible to dedicate extended periods to a single patient [46]. However, Davidson et al. indicate that considerable time is spent with patients during consultations. But patients are aware of the rush felt by HCPs during consultations, which leads to lower satisfaction levels and can even lead to discontinuation of AET [44].

#### 3.3.11 Synthesized finding 3.2: ongoing support

Ongoing support from HCPs and consistency in communication and care delivery are important. Due to the demanding and increasing care burden, many patients using AET are referred back to general practitioners for follow-up care. This leads to discontinuity, feelings of abandonment, and early stopping of AET [48]. To counter this, it can be recommended to schedule regular follow-up visits to reassess and reinforce treatment plans, which may help maintain adherence and reduce the likelihood of early treatment discontinuation [47].

Several studies assign the role of a consistent point of contact to a specialized nurse, as highlighted by Roche and Miaskowski. This is because nurses play a crucial role in reinforcing patient education, building trust, and gathering feedback, which helps HCPs assess adherence to AET. Their approachable nature and ability to manage adverse events by communicating with patients and HCPs enhance patient care [47]. Therefore, a more critical role for patient navigators, such as nurses, seems necessary, as some patients may be more likely to open up and share their concerns with them than physicians [57].

Frequent and well-structured physician communication significantly improves medication adherence (p ≤ 0.05) [58]. This aligns with the results that communication delays can hinder adherence and that regular monitoring and feedback are key to maintaining it [54,57].

## 4 Discussion

This systematic review aimed to explore key aspects of effective communication to enhance medication adherence to AET in breast cancer patients. Three synthesized findings comprising subcategories were identified: 1) Content of communication, with three subcategories: discussions on the importance and necessity of AET and adherence, AET-related side effects, and strategies to adhere to AET; 2) Process of communication, with four subcategories: building a trusting relationship, fostering open and mutual communication, engaging patients, and aligning with patients; and 3) Contextual preconditions for communication, with two subcategories: time constraints and ongoing support.

The finding ‘content of communication’ emphasizes that women feel the need to initiate conversations about AET side effects that HCPs may downplay, potentially leading to non-adherence. Better guidance on managing side effects, along with the supportive role of nurses, is essential for improving adherence to AET. Conversations frequently remain superficial, avoiding sensitive topics like sexual dysfunction and menopausal symptoms. This lack of open communication creates barriers to effective care. A study emphasizes that inadequate discussion of sexual health lowers AET adherence [59]. Addressing these disparities requires more resources for psychosocial aspects of cancer survivorship and encourages open discussions with oncologists [60]. Timely, effective, and repeated communication is crucial for improving adherence.

The finding ‘process of communication’ encompasses various communication strategies. Firstly, building a trusting relationship, characterized by empathy, non-judgmental support, and respect for patient perspectives, is vital for fostering open and mutual communication and improving adherence. Additionally, engaging patients in bidirectional communication empowers them, helping them mentally prepare for side effects and remain committed to treatment. Finally, aligning information with patients' needs using clear, simple language ensures they understand treatment and are more likely to continue therapy. These communication strategies appear effective in supporting medication adherence. Hence, HCPs must be aware of what these effective communication strategies entail about medication adherence. We defined 'being aware' as having knowledge and recognizing the existence or importance of communication and adherence. Discussing medication adherence may be integrated into regular follow-up consultations, meaning it does not need to be a separate, time-consuming activity.

The finding ‘contextual preconditions’ highlights that time constraints during consultations pose a significant challenge for HCPs, as they often cannot dedicate sufficient time to their patients. Patients are aware of this rushed atmosphere, which can lead to lower satisfaction and, in some cases, early discontinuation of AET. To address the challenge of time constraints, a potentially cost-effective strategy is the use of anti-hormonal decision aids [[61], [62], [63]]. These tools help patients make informed decisions about AET adherence prior to their consultation with a medical oncologist or nurse practitioner. By using this tool beforehand, patients can prepare specific questions, making the discussion during the consultation more focused and efficient. This approach might save time during follow-up consultations and ensure decisions are made thoughtfully and promptly [62]. However, further studies are needed to better understand the impact of decision aids on populations with lower health literacy or low socioeconomic status, since these factors influence decision-making outcomes [62,64].

Ongoing support from HCPs is essential for maintaining medication adherence. Our findings already suggest that nurses, in particular, play a key role in providing continuous support throughout the care journey, educating patients, and managing adverse events, thereby enhancing patient care and adherence. Their involvement in follow-up care enables them to optimize these processes, ultimately improving the patient's quality of life. This is further supported by Kahn et al., who found higher rates of ongoing AET use among patients with a single HCP primarily responsible for follow-up cancer care compared to those without a designated primary follow-up provider (P = 0.0066) [65]. Building on this, the nurse-patient relationship is central to delivering high-quality care. As the ‘Fundamentals of Care’ framework outlines, establishing trust is the initial step and must be maintained through continuous engagement. Anticipating patient needs is also a key element that empowers nurses to support patients effectively. The quality of this relationship is dynamic and continuously assessed and adjusted as needed, ultimately improving adherence and overall care outcomes [66].

### 4.1 Strengths, limitations and meta-biases

A key strength of this review was the use of a convergent integrated mixed-methods approach, which enabled the synthesis of qualitative and quantitative research, ensuring a comprehensive overview that captured all relevant findings [36]. Another strength was the close collaboration of all research team members (MS, SV, LM, LS). To ensure precision, two researchers (MS, SV) independently conducted all critical review steps, with regular consultations involving the entire team. However, a few limitations should be acknowledged. For example, most studies were conducted in the USA and Canada, where healthcare systems are organized and financed differently and may not be directly comparable to the typically more standardized and publicly funded systems in Western Europe. While we have identified and formulated certain strategies, some subpopulations, such as patients with limited access to care, low socioeconomic status, or specific cultural or ethnic backgrounds, may not be adequately represented. This could affect the applicability of our findings in current practice and highlights the need for further research across diverse healthcare contexts. The exclusion of studies not published in English or Dutch may also limit the scope of evidence. Lastly, to enhance transparency and ensure the reliability of our findings, we have reported the quality assessment process of the included studies. We recognize that low-quality studies may affect the conclusions. Still, despite these limitations, we have carefully reviewed and combined the evidence to ensure our findings are based on the most reliable and consistent results.

### 4.2 Implications for clinical practice and future research

The findings of this study suggest several practical implications for improving medication adherence. Firstly, HCPs need to be aware of what effective communication about medication adherence entails. To become more aware, HCPs can also be trained in communication strategies tailored to address patient adherence. A meta-analysis report that providing physicians with communication training significantly improves patient adherence, highlighting that such investment can substantially enhance treatment outcomes by increasing the likelihood of patients following medical advice [67]. King and Hoppe (2013) also advocate for high-intensity training interventions to improve patient-centred communication skills in medical encounters [68]. Secondly, HCPs and healthcare systems must enhance communication about the importance of AET, clearly explain its purpose and benefits, involve patients in shared decision-making, address side effects with coping strategies, and ensure equitable delivery of survivorship care, including adherence assessment, whilst fully recognizing their role in follow-up care [14,59]. Lastly, nurses play a crucial role in providing consistent, ongoing support throughout the care journey, as they can offer long-term guidance and assistance, which enhances both patient care and adherence. To address the challenge of time constraints HCPs may face, decision aids could be implemented to streamline follow-up consultations. In line with this, King and Hoppe (2013) support the notion that consultation time can decrease when HCPs improve their ability to ask and respond to patient questions, suggesting that better communication skills can enhance efficiency and quality of care [68].

Several implications for future research can be made. This includes exploring patient experiences in consultations, focusing on both the process and content of communication, including contextual preconditions. Additionally, observing patient-provider interactions can offer insights into (non-)verbal communication. Lastly, examining HCPs’ experiences will help assess feasibility and identify additional communication strategies.

### 4.3 Conclusion

This systematic review explores key aspects of effective communication to enhance medication adherence to AET in breast cancer patients. It offers valuable insights for HCPs to support medication adherence, highlighting the importance of effective communication between HCPs and patients. Key barriers include superficial conversations about side effects, discomfort in discussing sexual health issues, and time constraints during consultations, all of which can lead to non-adherence. Strategies such as building trust, fostering open and mutual communication, engaging in bidirectional communication, aligning information with patients' needs and utilizing tools like decision aids can empower patients, streamline consultations, and ensure comprehensive, patient-centred care. Nurses are vital in providing continuous support and addressing adverse events over time. These efforts collectively optimize appropriate follow-up care, enhancing patients’ quality of life.

## CRediT authorship contribution statement

**M.A.A. Smits:** Writing – original draft, Visualization, Validation, Project administration, Methodology, Investigation, Formal analysis, Data curation, Conceptualization. **L.H. Mammatas:** Writing – review & editing, Validation, Supervision, Methodology, Formal analysis, Conceptualization. **L. Schoonhoven:** Writing – review & editing, Supervision, Conceptualization. **S.C.J.M. Vervoort:** Writing – review & editing, Validation, Supervision, Methodology, Investigation, Formal analysis, Conceptualization.

## Ethical approval

No ethical approval was required.

## Funding

This research did not receive any specific grant from funding agencies in the public, commercial, or not-for-profit sectors.

## Declaration of competing interest

The authors declare no potential conflicts of interest with respect to the research, authorship, and/or publication of this article.
